# Improved Metathesis Lifetime: Chelating Pyridinyl-Alcoholato Ligands in the Second Generation Grubbs Precatalyst

**DOI:** 10.3390/molecules19055522

**Published:** 2014-04-29

**Authors:** Jean I. du Toit, Margaritha Jordaan, Carlijn A. A. Huijsmans, Johannes H. L. Jordaan, Cornelia G. C. E. van Sittert, Hermanus C. M. Vosloo

**Affiliations:** Research Focus Area for Chemical Resource Beneficiation, Catalysis and Synthesis Research Group, North-West University, Potchefstroom 2520, South Africa; E-Mails: 12317624@nwu.ac.za (J.I.T.); rita.jordaan@telkomsa.net (M.J.); 10063552@nwu.ac.za (C.A.A.H.); johan.jordaan@nwu.ac.za (J.H.L.J.); cornie.vansittert@nwu.ac.za (C.G.C.E.S.)

**Keywords:** second generation grubbs precatalyst, pyridinyl-alcoholato ligand, 1-octene metathesis, lifetime

## Abstract

Hemilabile ligands can release a free coordination site “on demand” of an incoming nucleophilic substrate while occupying it otherwise. This is believed to increase the thermal stability and activity of catalytic systems and therefore prevent decomposition via free coordination sites. In this investigation chelating pyridinyl-alcoholato ligands were identified as possible hemilabile ligands for incorporation into the second generation Grubbs precatalyst. The *O*,*N*-alcoholato ligands with different steric bulk could be successfully incorporated into the precatalysts. The incorporation of the sterically hindered, hemilabile *O*,*N*-ligands improved the thermal stability, activity, selectivity and lifetime of these complexes towards the metathesis of 1-octene. A decrease in the activity of the second generation Grubbs precatalyst was additionally observed after incorporating a hemilabile *O*,*N*-ligand with two phenyl groups into the system, while increasing their lifetime.

## 1. Introduction

**Figure 1:**
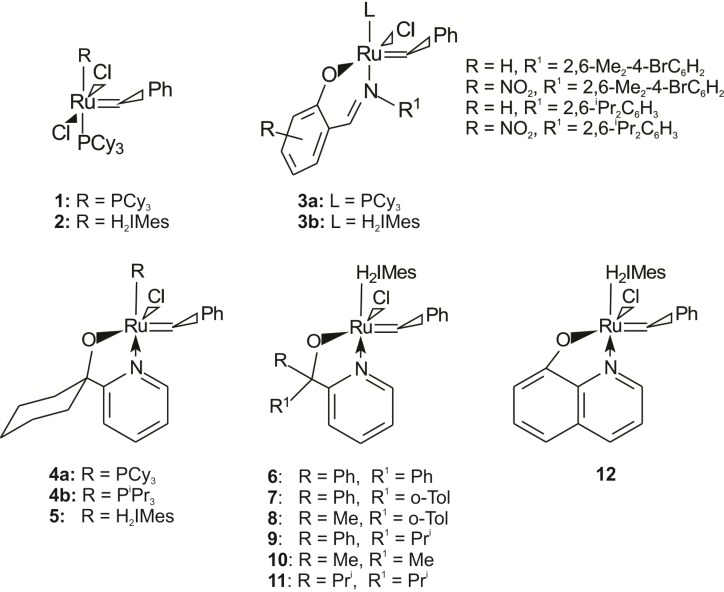
Grubbs-type precatalysts.

In the last decade the development of alkene metathesis catalysts has made significant progress with the design of catalytic systems to improve one or all of the following aspects: (i) catalyst efficiency and activity, (ii) substrate scope and selectivity and (iii) the removal of metal impurities and catalyst recycling.[1] The ruthenium carbene complexes developed by the Grubbs group, **1** and **2** (Figure 1), remain of great importance due to their high activity and tolerance towards polar functional groups [2,3].

**Figure 2:**
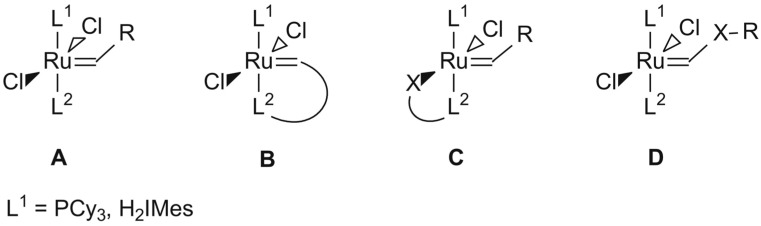
Design concepts for thermally switchable initiators [7,8,9,10,11,12,13].

The development of new classes of ligands bearing donor atoms other than phosphorus was inspired by the fact that phosphine ancillary ligands undergo degradation (e.g., P-C bond cleavage, oxidation to phosphine oxides, *etc.*) during catalysis, especially under thermal conditions, which limits the lifetime of **1** [4,5,6]. The incorporation of an N-heterocyclic carbene (NHC) into **1**, provided precatalyst **2** with improved activity and lifetime, despite its low selectivity at elevated temperatures. The limited thermal stability of **1** and **2** is believed to be due to the fact that PCy_3_ is very labile at room temperature resulting in fast decomposition of the precatalyst. This has motivated several groups to design new thermally switchable initiators (Figure 2) with chelating ligands, where L_2_ is either attached to the carbene (motif **B**, Hoveyda-type precatalysts [7,8,9,10]) or via X (motif **C**, [11,12,13] where X is for example an oxygen) to the central ruthenium atom.

For motif **C** Grubbs [12] and later Verpoort [14,15] introduced bidentate O,N-chelated Schiff base ligands on **1** and **2** to give **3a** and **3b** (Figure 1), respectively. The combination of a Schiff-base ligand with an NHC-ligand to produce **3b**, increased the thermal stability and activity of these complexes towards ROMP and RCM [11,13,14]. Another example for motif **C** is the first and second generation ruthenium benzylidene complexes synthesized by Herrmann and coworkers [11] bearing a hemilabile pyridinyl alcoholato ligand (**4**) (Figure 1).

The NHC catalytic systems showed low activity for ROMP at room temperature due to their resting state stabilization [11]. The catalytic activity of these systems increased with an increase in temperature, which was comparable to **2**. The first generation system **4a** has been shown to be active for 1-octene metathesis at 60 °C, with a 1-octene/Ru molar ratio of 9000 in a neat solution [22]. Van der Schaaf *et al.* [16] have patented a number of symmetrical and unsymmetrical *O*,*N*-chelated ruthenium alkylidene systems with a tri-isopropylphosphine ligand with no reported catalytic activity. *O*,*N*-chelated pyridinyl alcoholato second generation ruthenium alkylidene precatalysts **6** and **10** [17,18,19], showing an extraordinary stickiness to silica, were reported for the cross-metathesis reaction of 5-decene with 5-hexenyl acetate [19]. The use of these complexes on a TLC plate for catalyst screening were also reported [18]. Slugovc and Wappel [20] have patented a number of *O*,*N*-chelated 8-quinolinolate ruthenium alkylidene systems (**12**). We have reported the *O*,*N*-chelated pyridinyl alcoholato second generation ruthenium alkylidene precatalyst **5** which was shown to be active towards the metathesis of 1-octene at higher temperatures in a neat solution at low Ru loadings [22]. The diphenyl substituted precatalyst **6** also showed very good catalytic lifetime in separation studies [21]. Here we report on the synthesis and characterization of a number of new pyridinyl alcoholato second generation ruthenium alkylidene complexes (**7**–**9**). The activity and lifetime in the metathesis reaction with 1-octene of these precatalysts were compared with that of known complexes (**2**,**5**,**6**,**10**–**12**) (Figure 1). Apart from complex **12**, all the complexes showed high activity, temperature stability and a long lifetime towards the self-metathesis of 1-octene, relative to **2**.

## 2. Results and Discussion

### 2.1. Synthesis and NMR Characterization of Second Generation O,N-Chelated Ruthenium Carbene Complexes

The procedure followed for the synthesis of precatalysts **5–12**, consisted of stirring **2** with the lithium salt of the corresponding alcohols at 35–40 °C for 2 days in THF [22].

As previously reported for **5** [22], the high solubility of LiCl in THF, which drove the reaction, may have caused the reactions to take longer than that reported by Denk *et al.* [11] for similar complexes.

The complexes were obtained in moderate yields as microcrystalline powders, but no suitable crystals for X-ray crystallographic analysis could be obtained through the slow diffusion of pentane into a saturated solution of the complexes in THF. For **7**, **8** and **9**, it was necessary to repeat the washing procedure twice to obtain a pure product. These complexes were obtained in lower yields (**7** = 16%, **8** = 27%, **9** = 49%) which are probably a result of the intensive purification procedure used. It was however noted that **12** was soluble in most of the solvents used for recrystallization, and was therefore obtained in low yield as an orange powder through slow vacuum condensation of pentane after sonificating the solution for 10 min.

The ^1^H-NMR chemical shifts of the carbene α-H signal (denoted H* in the structures) and pyridine α-H signal (denoted as H^†^ in the structures) for precatalysts **5**–**12** are summarized in Table 1.

**Table 1:** Selected ^1^H-NMR signals of the second generation hemilabile complexes as compared to the free *O*,*N*-ligands and **2**.

Catalyst	δ_H__*_	δ_H_^†^^a^	δ_H_^†^^b^
2	19.19, s	-	-
5	17.96, s	9.55, d	8.48, d
6	17.18, s	9.67, d	8.50, d
7	17.32, s	9.75, d	8.59, d
8	17.31, s	9.87, d	8.54, d
9	17.33, s	9.39, d	8.44, d
10	17.82, s	9.15, d	8.49, d
11	18.52, s	9.65, d	8.52, d
12	18.25, s	7.75, d	8.77, d

^a^ carbene α-H signal (denoted H*) and pyridine α-H signal (denoted as H^†^) of the hemilabile complexes; ^b^ pyridine α-H signal (denoted as H^†^) of the pyridinyl carbinol ligands.

Similar to the first generation hemilabile complexes [22], a strong upfield shift is observed in the H_α_ signals of the second generation hemilabile complexes. Relative to **2**, the H_α_ signals also appeared as singlets. As expected no ^31^P-NMR resonances were observed, since no phosphorous group should be coordinated to the Ru-centre after substitution with the *O*,*N*-chelated ligands. Additionally a downfield shift in the pyridine α-H signal was observed for complexes **5**–**12**, indicating that the electronic environment of the ligand has changed. This implies that the N-atom has coordinated to the Ru-centre, since a downfield shift of a proton resonance signal is indicative of reduced electron density of the attached C-atom [23].

### 2.2. Metathesis of 1-Octene with Different Precatalysts

During the metathesis reaction of 1-octene a mixture of products can form (Table 2). This mixture is obtained due to alkene isomerisation and the continued formation of new metathesis products via self- and cross-metathesis [24].

**Table 2:** Possible reactions of 1-octene in the presence of metathesis catalysts [24].

Reaction	Substrate ^a^	Products ^a^	
Primary metathesis			
*- Self-metathesis*	C=C_7_	C=C + C_7_=C_7_	(PMP) ^b^
*- Isomerisation*	C=C_7_	C_2_=C_6_ + C_3_=C_5_ + C_4_=C_4_	(IP) ^c^
Secondary metathesis			
*- Cross-metathesis*	C=C_7_ + C_2_=C_6_	C_2_=C_7_ + C=C_6_ + C=C_2_ + C_6_=C_7_	(SMP) ^d^
*- Self-metathesis*	C_2_=C_6_	C_2_=C_2_ + C_6_=C_6_	

^a^ Hydrogens are omitted and geometrical isomers not shown for simplicity; ^b^ Primary metathesis products (PMP) refers to the self-metathesis products of 1-octene, *i.e.*, C_7_=C_7_ and C=C; ^c^ Isomerisation products (IP) refers to the double bond isomerisation reaction of terminal to internal alkenes; ^d^ Secondary metathesis products (SMP) refers to the metathesis of the isomerisation products of 1-octene.

The optimum working temperature whereby **5** gives high activity while retaining a high degree of selectivity towards PMP formation with a limited amount of SMP and IP formation was reported to be 60 °C [22]. It was therefore decided to investigate the catalytic activity of **6**–**12** at 60 °C, with 1-octene/Ru molar ratio = 9000 in the absence of any solvent.

In Table 3 the 1-octene metathesis activity and selectivity of the second generation hemilabile precatalysts are compared to **2** after 420 min and in Table 4 after 10 h up to 1.5 days. For the calculation of the percentage of 1-octene, PMP, IP or SMP the following formula was used: %n_x_ = (n_x_/n_tot_) × 100. Where %n_x_ = mol percentage of 1-octene, PMP, IP or SMP; n_x_ = mol of 1-octene, PMP, IP or SMP and n_tot_ = sum of mol of 1-octene, PMP, IP and SMP. The turnover number (TON) in this study was calculated using the formula (%PMP × (oct/Ru))/100 with oct/Ru the 1-octene/Ru molar ratio at t = 0 and the turnover frequency (TOF) as TON h^−1^.

**Table 3:** Catalytic activity and selectivity of the precatalysts at 60 °C (1-octene/Ru = 9000, no solvent) after 420 min.

Precatalyst	%PMP ^a^	%SMP ^b^	%IP ^c^	%S ^d^	TON	TOF
2	80.6	3.7	0	96.3	7254	1036
5	80.4	13	0	85.5	7236	1034
6	35.6	0.9	0	99.1	3204	458
7	20	3.7	0.9	95.3	1800	257
8	80.6	18.6	0.1	80	7254	1036
9	48	6.8	0.5	92.7	4320	617
10	74	21.8	0	78.2	6660	951
11	86.5	12.4	0	86.1	7785	1,112
12	0.4	0	0.2	99.8	36	5

^a^ C=C + C_7_=C_7_; ^b^ C_2_=C_7_ + C=C_6_ + C=C_2_ + C_6_=C_7_; ^c^ C_2_=C_6_ + C_3_=C_5_ + C_4_=C_4_; ^d^ Selectivity towards PMP.

**Figure 3:**
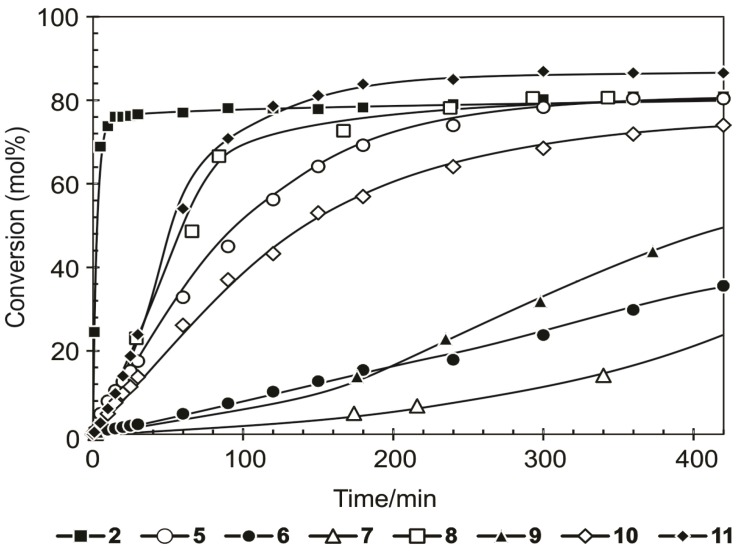
The formation of PMPs during the metathesis of 1-octene in the presence of **2**, **5**–**11** at 60 °C and a 1-octene/complex molar ratio of 9000.

The lifetime and activity of the second generation precatalysts have been improved regardless of the slow initiation rate of these systems compared to **2** at 60 °C. However, an approximate 10%–20% decrease in the selectivity of **5**, **8**, **10** and **11** towards the formation of PMPs was evident after 420 min compared to **2**, while the selectivity of **6** and **12** slightly increased, and **7** and **9** showed similar selectivity. After 20 h (Table 4) all the precatalysts still showed activity towards the metathesis of 1-octene, *i.e.*, between 80%–90% PMP for **5**, **8**, **9** and **10**, and 86.5% and 94% PMP for **11** and **7** respectively, while **2** (82% PMP) is inactive after *ca.* 7 h. Therefore a 4%–10% increase in PMP formation for **5** and **10** was observed after 20 h compared to a 35%, 40% and 74% increase for **6**, **9 ** and **7** respectively, indicating the high stability and activity of these complexes. The SMP formation for all the precatalysts remained almost unchanged with only a 1%–5% increase leading to a *ca.* 2% decrease in the selectivity of **5**, **6** and **10**, and a 4% decrease for **9** towards the formation of PMPs. Furthermore, a 5%–10% increase in TON of **5** and **10** was observed after 20 h. However, an 84%, 97% and 370% increase in cumulative TON of **9**, **6** and **7** respectively, was observed after 20 h. Although the observable IPs for all the complexes remained below 0.2%, an increase to a maximum within the first 5–10 min was followed by a sharp decrease to *ca.* 0% within 10–30 min as the reaction proceeded, most probably due to cross-metathesis of the IPs to form SMPs. The major SMP product was found to be tridecene (C_13_) for all the systems, which is mainly as a result of the cross-metathesis of 1- and 2-octene. However, **12** showed very low activity towards the formation of PMPs, only achieving 0.4% PMP formation and were not investigated further. The formation of PMPs during the metathesis reactions after 420 min is shown in Figure 3.

**Table 4:** Catalytic activity and selectivity of the precatalysts at 60 °C (1-octene/Ru = 9000, no solvent) after 10 h up to 1.5 days.

Precatalyst	%PMP ^a^	%SMP ^b^	%IP ^c^	%S ^d^	t (min) ^e^	TON	TOF
2	81.6	3.6	0	96.4	1200	7344	367
5	87.8	12.2	0	84.4	1246	7902	381
6	70.3	2.8	0	97.2	1200	6327	316
7	94	5.5	0	94.5	2098	8460	242
8	80	19.9	0	80.1	630	7200	686
9	88.2	11.5	0	88.5	1121	7938	425
10	82.3	17	0	75.8	1246	7407	357
11	86.5	12.4	0	86.1	1246	7785	375
12	0.4	0	0.2	99.8	1200	36	2

^a^ C=C + C_7_=C_7_; ^b^ C_2_=C_7_ + C=C_6_ + C=C_2_ + C_6_=C_7_; ^c^ C_2_=C_6_ + C_3_=C_5_ + C_4_=C_4_; ^d^ Selectivity towards PMP; ^e^ Reaction time to maximum conversion.

### 2.3. Stability of Different Precatalysts

**Figure 4:**
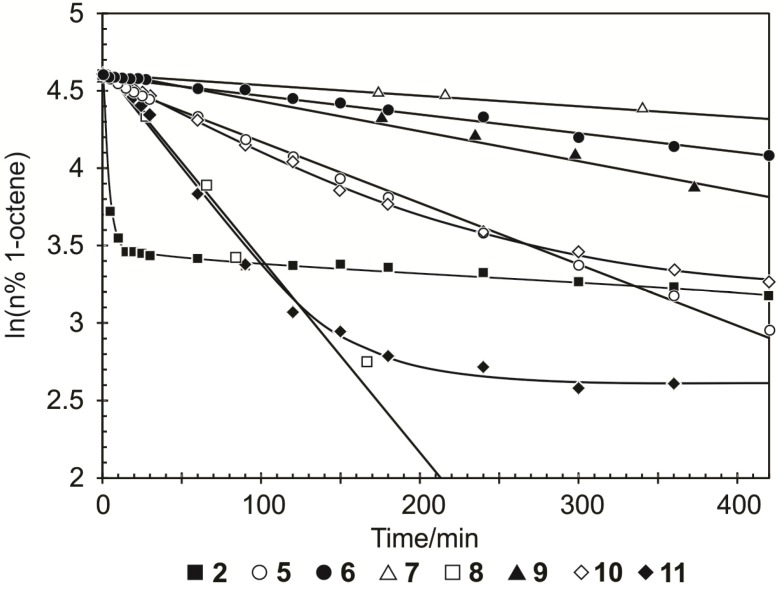
Logarithmic plots for the precatalysts at 60 °C and a 1-octene/Ru molar ratio = 9000.

To obtain an idea of the stability of the different precatalysts the definition and approach of Grubbs and coworkers [25] were used. The stability of the Ru precatalysts is defined as the lifetime of the catalytic species during the course of the reaction and can be monitored by the loss of activity. They used plots of ln([starting material]) *versus* time as an illustration of the relative stabilities of Ru precatalysts. A linear plot indicates a reaction with pseudo-first-order rate kinetics while a curved plot points towards catalyst decomposition. The relative stability of the precatalysts is compared in Figure 4 by plotting the ln(n% substrate) *versus* time.

The linear plots of **5**, **6**, **7**, **8** and **9** compared to the curved plots of **2**, **10** and **11**, indicates that the aforementioned systems are more stable [25]. Precatalyst **2** was not active upon further addition of 1-octene after 3 h while **6** showed reduced activity after 3 further additions of 1-octene [21]. However, despite the high thermal stability of **5** and **8** low selectivities are displayed towards PMP formation. On the other hand, high selectivity and stability is observed for **6**, **7** and **9** despite its slow activity.

### 2.4. Optimization of Reaction Conditions for **7**, **8** and **9**

Optimization reactions of the newly synthesized precatalysts **7**, **8** and **9**, in comparison with **6**, were performed by changing the 1-octene/Ru molar ratio, as well as the reaction temperature. When the complexes are used at different molar ratios at 60 °C the same overall trends are observed for the reaction time to maximum conversion and TON (Table 5), *i.e.*, it increases with an increase in 1-octene/Ru molar ratio. There is however a difference in the trends observed for PMP and SMP formation with an increase in molar ratio. Precatalyst **6** shows an increase in both PMP and SMP. However, **7** and **8** show an increase in PMP formation (decrease in SMP) while **9** shows a decrease in PMP (increase in SMP).

**Table 5:** Catalytic activity and selectivity of **7**, **8** and **9** at 60 °C and different 1-octene/Ru molar ratios.

Precatalyst	1-octene/Ru	%PMP ^a^	%SMP ^b^	t (min) ^c^	TON	TOF
6	7000	30.9	1.1	420	2165	309
	9000	70.3	2.8	1200	6327	316
7	6500	93.2	6.7	1875	6058	194
	9000	94.0	5.5	2098	8460	242
8	4500	77.0	20.6	313	3465	664
	9000	80.0	19.9	595	7200	726
	10,700	81.6	17.1	481	8731	1,089
	12,000	88.0	11.1	970	10,560	653
9	9000	88.2	11.5	1265	7938	377
	12,000	81.0	18.1	1244	9720	469
	14,000	81.9	18.0	1752	11,466	393

^a^ C=C + C_7_=C_7_; ^b^ C_2_=C_7_ + C=C_6_ + C=C_2_ + C_6_=C_7_; ^c^ Reaction time to maximum conversion.

**Figure 5:**
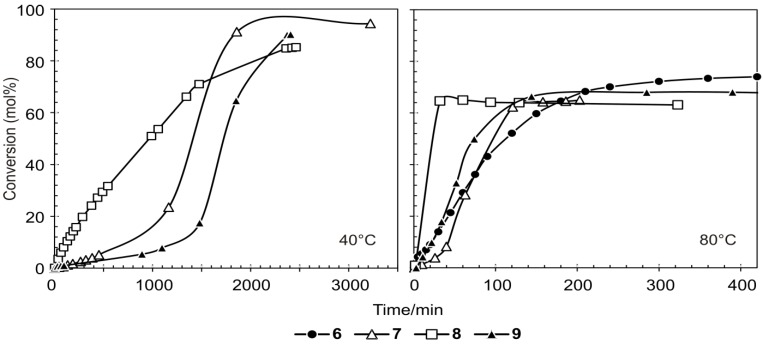
The formation of PMPs during 1-octene metathesis in the presence of **6**, **7**, **8** and **9** at 40 °C and 80 °C and a 1-octene/Ru molar ratio = 9000.

When the precatalysts are used at different reaction temperatures and the same 1-octene/Ru molar ratio = 9000 the same overall trends are observed (Table 6). For all the precatalysts an increase in reaction temperature leads to a faster conversion (Figure 5), a decrease in PMP formation and TON, while an increase in SMPs and TOF is observed. The initial increase of PMPs follows a linear trend with increasing slopes as the temperature increases which is similar for all the precatalysts except for **9** at 40 °C. For **9** a slow initiation of about 1400 min is observed at 40 °C (Figure 5).

**Table 6:** Catalytic activity and selectivity of **6**, **7**, **8** and **9** at different temperatures and an 1-octene/Ru molar ratio = 9,000.

Precatalyst	T (°C)	%PMP ^a^	%SMP ^b^	t (min) ^c^	TON	TOF
6	40	-	-	420	-	-
	60	70.3	2.8	1200	6327	316
	80	74.0	11.5	420	6661	952
	120	10.7	77.8	1256	963	46
7	40	95.4	3.6	3224	8586	160
	60	94.0	5.5	2098	8460	242
	80	64.9	34.4	203	5841	1726
8	40	85.2	10.5	2463	7668	187
	60	80.0	19.9	595	7200	726
	80	63.1	35.6	323	5679	1055
9	40	90.2	9.4	2410	8118	202
	60	88.2	11.5	1265	7938	377
	80	64.4	35.4	981	5796	354

^a^ C=C + C_7_=C_7_; ^b^ C_2_=C_7_ + C=C_6_ + C=C_2_ + C_6_=C_7_; ^c^ Reaction time to maximum conversion.

An increase in temperature results in a 20% average decrease in PMP formation, with a simultaneous 20% average increase in SMP formation within 60 min for all four precatalysts. This indicates that the isomerisation activity of the precatalysts increased with an increase in temperature, which led to an increase in SMP formation due to the cross-metathesis of the IPs. The isomerisation can probably occur either by reversible allyl-hydride formation or due to the decomposition of the precatalyst to form a Ru-hydride intermediate [26]. These mechanisms have been previously explained in much detail for the Grubbs carbenes and are also applicable to our system [26].

### 2.5. Catalytic Lifetime Studies of Precatalysts **7**, **8** and **9**

To obtain an idea of the extent of the catalytic lifetime of **7**, **8** and **9** 1-octene (20 mL) was added consecutively when most of the 1-octene was converted in the previous step. Table 7 shows the time it takes for the precatalysts to convert the maximum amount of 1-octene to PMPs and SMPs.

**Figure 6:**
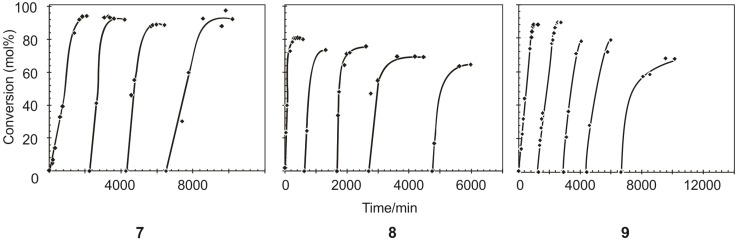
The formation of PMPs during metathesis in the presence of **7, 8** and **9** with consecutive additions of 1-octene at 60 °C and an initial 1-octene/Ru molar ratio of 9000.

In general it was found that the activity, selectivity and TON decreases after each new addition of 1-octene. With each addition the reaction time to maximum conversion increases. This may mainly be due to the 1-octene/Ru molar ratio increasing, *i.e.*, the precatalyst loading decreasing, with each addition. Furthermore, the average % PMP formation for **8** and **9** was 73% and 81% respectively after a fifth addition of 1-octene and that of **7** was 92% after a fourth addition. A cumulative TON of the three precatalysts with an initial precatalyst loading of 1:9000 with consecutive additions of 1-octene is 33,039 for **7**, 32,634 for **8** and 36,576 for **9**. The formation of the PMPs for the different precatalysts is shown in Figure 6. Similar trends with a steady decrease in PMP formation for **8** and **9** are observed.

The precatalysts remained active for days after the final addition of the 1-octene. Precatalyst **7** was active for a further 7 days (10,325 min), **8** for 4 days (5,976 min) and **9** for 8 days (11,827 min). Precatalyst **6** was active for about 2 days according to literature [21]. It is clear that all three precatalysts show a significant improvement in lifetime when compared to **2**.

**Table 7:** Catalytic activity and selectivity of **7**, **8** and **9** with consecutive additions of 1-octene at 60 °C and an initial 1-octene/Ru molar ratio = 9000.

Precatalyst	Addition	%PMP ^a^	%SMP ^b^	%S ^c^	t (min) ^d^	TON	TOF
7	1	94.3	5.6	94.4	2190	8487	233
	2	91.8	8.2	91.8	2067	8262	240
	3	88.8	11.1	88.9	2246	7992	213
	4	92.2	7.6	92.4	3822	8298	130
8	1	80.0	19.9	80.1	630	7200	686
	2	73.4	26.1	73.8	1039	6606	381
	3	75.5	23.8	76.0	1024	6795	398
	4	69.1	30.6	69.3	2036	6219	183
	5	64.6	33.6	65.8	1247	5814	280
9	1	88.2	11.5	88.5	1298	7938	367
	2	89.5	9.4	90.5	1632	8055	296
	3	78.2	13.8	85.0	1484	7038	385
	4	71.7	6.2	92.0	2246	6453	172
	5	78.8	9.8	88.9	5167	7092	82

^a^ C=C + C_7_=C_7_; ^b^ C_2_=C_7_ + C=C_6_ + C=C_2_ + C_6_=C_7_; ^c^ Selectivity towards PMP, ^d^ Reaction time to maximum conversion.

## 3. Experimental Section

### 3.1. Reagents and Solvents

Diethyl ether (b.p. 34.6 °C, Labchem, Johannesburg, South Africa), THF (b.p. 66 °C, Saarchem, Johannesburg, South Africa) and toluene (b.p. 110–111 °C, Merck, Johannesburg, South Africa) were distilled under N_2_ from sodium with benzophenone as indicator. Pentane (b.p. 35–36 °C, Labchem) was distilled under N_2_ from CaH_2_. THF and toluene were stored over 4 Å mol sieves. Diethyl ether and pentane were used directly from the distillation. The 1-octene (98%, Sigma-Aldrich, Johannesburg, South Africa) was purified by filtering the solution through an alumina column after the alumina was dried overnight at 70 °C. The solution was stored over 3 Å mol sieves. *n*-Butyl lithium, 2-bromopyridine (99%), isobutyrophenone, 2-methylbenzophenone, 2'-methylacetophenone, cyclohexanone, benzophenone and the 2nd generation Grubbs precatalyst were purchased from Aldrich and used as received. tert-Butyl hydroperoxide (5.5 M in decane) and nonane (99%) were purchased from Fluka (Johannesburg, South Africa) from which the first mentioned was stored over 4Å mol sieves. All gases used during this study were supplied by Afrox (Johannesburg, South Africa).

### 3.2. Characterization Techniques

For the characterization of the synthesized ligands and complexes, the following apparatus were used. The melting points of the ligands were determined on a Büchi B-540 melting point apparatus. Infra-red spectra of all the compounds were obtained on a Nicolet FTIR 550 infrared spectrophotometer (IR) [a KBr pellet was prepared of all samples (0.005 g sample mixed with 0.28 g dry KBr) and analyzed using 10 scans over a wave number range of 400–4000 cm^−1^]. The molecular masses of the ligands were obtained on a Micromass Autospec mass spectrometer and the molecular masses of the complexes on a Bruker Ultraflex III TOF/TOF (TOFMS) [samples were prepared by mixing 2 µL (1 mg/mL in CHCl_3_) with 20 µL DCTB (20 mg/mL in THF); 1 µL was placed on a Bruker ground steel target plate before analysis]. A Bruker MicroTOF-Q II mass spectrometer was used for the determination of high resolution masses. For the determination of the purity as well as the mass of the alcohols an Agilent Technologies 6890N Gas Chromatograph equipped with a Phenomenex ZB-1 column (30 m × 320 μm × 1 μm), a 7683B autoinjector and 5973 Mass Selective Detector was used. Nuclear magnetic resonance spectra (^1^H, ^13^C and ^31^P) of all compounds were obtained on a Varian 300MHz and a Bruker Avance II 600MHz spectrometer (NMR) [20 mg sample was dissolved in 1.5 mL CDCl_3_; the samples of the complexes were prepared under inert conditions using freeze dried CDCl_3_].

### 3.3. General Procedure for the Preparation of Lithium Salts

For the preparation of lithium salts, the alcohol (4 mmol) was dissolved in THF (40 mL) and *n*-butyl lithium (1.8 mL, 4 mmol, 2.5 M) was added dropwise to the reaction mixture under inert (Ar) and very dry conditions. This reaction was carried out at room temperature, so it was important to add the *n*-butyl lithium slowly due to the exothermal character of the reaction. After 2 h of stirring the solution became slightly yellow and no precipitate formed. When the THF was removed under reduced pressure a yellow solid was obtained. The solid was washed with dry pentane (5 × 20 mL) forming a white powder. The white powder was dried under vacuum and used without further characterization or purification, due to its sensitivity to air.

### 3.4. General Procedure for the Synthesis of the Second Generation Precatalysts [22]

A solution of lithium alcoholate (0.63 mmol, 0.090 g) in THF (5 mL) was added dropwise to a solution of **2** (0.55 mmol, 0.470 g) in THF (5–10 mL) under inert (Ar) and very dry conditions. The reaction was stirred at 30–40 °C for 60min with a colour change from brown to various shades of green. The progress of the reaction was monitored by TLC (Merck Silica gel 60 F254; eluent hexane–ethyl acetate = 1:2) to determine if the starting complex was being consumed. The reaction mixture was stirred until the TLC indicated that the starting complex was not present any more. After removing the solvent under vacuum the residue was dissolved in a minimal amount of toluene. The lithium chloride was then removed by filtration via a syringe filter and the solution concentrated to a volume of *ca.* 1 mL. The complexes were obtained by adding 10–15 mL cold pentane onto the filtrate and placing it in the fridge for 8 h or by immediate sonification for 10 min resulting in the immediate formation of a precipitate. After removal of the pentane via syringe or filtration, the desired complex was washed with cold pentane to produce analytically pure microcrystalline powders.

*Benzylidene-chloro(1,3-bis-(2,4,6-trimethylphenyl)-2-imidazolidinylidene)-[1-(2′-pyridinyl)-1,1-diphenylmethanolato]ruthenium* (**6**). Light green microcrystalline powder (91% yield). ^1^H-NMR (300 MHz, CDCl_3_): δ = 17.18 (s, 1H, Ru=CHPh), 6.43 (d, 2H, *ortho* H of C_6_H_5_), 7.19 (dd, 1H, *para* H of C_6_H_5_), 6.77 (dd, 2H, *meta* H of C_6_H_5_), 9.67 (d, 1H, *ortho* H of C_5_H_4_N), 7.19 (dd, 1H, *para* H of C_5_H_4_N), 7.04 (dd, 1H, *meta* H of C_5_H_4_N), 6.64 (d, 1H, *meta* H of C_5_H_4_N), 6.71 and 6.99 (s, 4H, *meta* H mesityl), 4.05 (bs, 4H, CH_2_ of NHC), 2.20, 2.30 and 2.65 (3s, 18H, CH_3_ mesityl), 7.12–7.19 (m, 10H, phenyl Hs of O,N-ligand); anal. C_46_H_47_ClN_3_ORu (794.42g/mol): C 69.55, H 5.96, N 5.29; calcd. for C 69.97, H 6.33, N 5.16.

*Benzylidene-chloro(1,3-bis-(2,4,6-trimethylphenyl)-2-imidazolidinylidene)-[1-(2′-pyridinyl)-1-phenyl-1-(2-tolyl)-methanolato]ruthenium* (**7**). Yield: 16%; ^1^H-NMR (600 MHz, CDCl_3_): δ_H_ 17.33 (s, 1H, Ru=CHPh), 9.76–9.53 (d, 1H, *ortho* of C_4_H_5_N), 7.10–7.08 (dd, 2H, *para* Hs of C_6_H_5_ and C_5_H_4_N), 7.08–6.99 (m, 13H, aromatic Hs of O,N ligand and mesityl), 6.68–6.55 (t, 2H, *meta* Hs of C_6_H_5_ and C_5_H_4_N), 6.44–6.36 (d, 1H, *meta* H of C_5_H_4_N), 6.36–6.16 (d, 1H, *ortho* of C_6_H_5_), 3.73–3.47 (m, 4H, m, Hs of NHC), 2.49–1.83 (3s, 18H, CH_3_ mesityl), 1.24 (s, 3H, tolyl CH_3_ of N,O ligand) ppm, Maldi-TOF: 807 *m/z*, [M^+^], HRESIMS *m/z* 807.2498 (calcd for C_47_H_48_ClN_3_ORu, 807.2533).

*Benzylidene-chloro(1,3-bis-(2,4,6-trimethylphenyl)-2-imidazolidinylidene)-[1-(2′-pyridinyl)-1-(2-tolyl)-1-methylmethanolato]ruthenium* (**8**). Yield: 27%; ^1^H-NMR (600 MHz, CDCl_3_): δH 17.32 (s, 1H, Ru=CHPh), 9.87–9.46 (d, 1H, *ortho* of C_4_H_5_N), 7.44–7.26 (d, 1H, *ortho* H of C_6_H_5_), 7.13–6.99 (m, 2H, *para* Hs of C_6_H_5_ and C_5_H_4_N), 6.97–6.72 (m, 8H, aromatic Hs of O,N ligand and mesityl), 6.70–6.57 (t, 2H, *meta* Hs of C_6_H_5_ and C_5_H_4_N), 6.33–6.00 (d, 1H, *meta* H of C_5_H_4_N), 4.24–3.78 (t, 4H, Hs of NHC), 2.60–2.36 (m, 18H, CH_3_ mesityl), 1.57 (s, 3H, CH_3_ of N,O ligand), 1.41–1.09 (m, 3H, tolyl CH3 of N,O ligand) ppm, Maldi-TOF: 745 *m/z* [M+], HRESIMS *m/z* 745.2500 (calcd for C_43_H_46_ClN_3_ORu, 745.2498).

*Benzylidene-chloro(1,3-bis-(2,4,6-trimethylphenyl)-2-imidazolidinylidene)-[1-(2′-pyridinyl)-1-phenyl-1-isopropylmethanolato]ruthenium* (**9**). Yield: 49%; ^1^H-NMR (600 MHz, CDCl_3_): δ_H_ 17.33 (s, 1H, Ru=CHPh), 9.39–9.38 (d, 1H, *ortho* H of C_4_H_5_N), 7.29–7.27 (d, 1H, *ortho* H of C_6_H_5_), 7.03 (m, 3H, aromatic Hs of O,N ligand), 6.94 (m, 2H, *para* Hs of C_6_H_5_ and C_5_H_4_N), 6.87–6.845 (m, 2H, *meta* Hs of mesityl), 6.59–6.57 (t, 2H, *meta* Hs of C_6_H_5_ and C_5_H_4_N), 4.08–4.00 (t, 4H, Hs of NHC), 2.72–2.12 (m, 18H, CH_3_ mesityl), 1.28–1.24 (m, 3H, *iso*-propyl CH_3_ of *O*,*N*-ligand), 0.65–0.64 (m, 3H, *iso*-propyl CH_3_ of *O*,*N*-ligand), 1.55–1.34 (t, 1H, *iso*-propyl CH of O,N-ligand) ppm, Maldi-TOF: 758 *m/z* [M^+^], HRESIMS *m/z* 758.2463 (calcd for C_43_H_47_ClN_3_ORu, 758.2454).

*Benzylidene-chloro(1,3-bis-(2,4,6-trimethylphenyl)-2-imidazolidinylidene)-[1-(2′-pyridinyl)-propane-2-olato]ruthenium* (**10**). Green microcrystalline powder (55% yield). ^1^H-NMR (300 MHz, CDCl_3_): δ = 17.82 (s, 1H, Ru=CHPh), 7.15 (d, 2H, *ortho* H of C_6_H_5_), 7.05 (dd, 1H, *para* H of C_6_H_5_), 6.85 (dd, 2H*, meta* H of C_6_H_5_), 9.15 (d, 1H, *ortho* H of C_5_H_4_N), 7.10 (dd, 1H, *para* H of C_5_H_4_N), 6.75 (dd, 1H, *meta* H of C_5_H_4_N), 6.60 (d, 1H, *meta* H of C_5_H_4_N), 6.70 and 6.85 (bs, 4H, *meta* H mesityl), 4.02 (m, 4H, CH_2_ of NHC), 2.15, 2.60 and 2.80 (s, CH_3_ mesityl), 1.20 (s, 6H, CH_3_ of *O*,*N*-ligand); anal. C_36_H_43_ClN_3_ORu (670.28 g/mol): C 64.51, H 6.47, N 6.27; calcd. for C 64.03, H 5.99, N 5.81.

*Benzylidene-chloro(1,3-bis-(2,4,6-trimethylphenyl)-2-imidazolidinylidene)-[1-(2′-pyridinyl)-2,4-dimethylpentan-3-olato]ruthenium* (**11**). Green microcrystalline powder (57% yield). ^1^H-NMR (300 MHz, CDCl_3_): δ = 18.52 (s, 1H, Ru=CHPh), 7.65 (d, 2H, *ortho* H of C_6_H_5_), 7.55 (dd, 1H, *para* H of C_6_H_5_), 7.35 (dd, 2H, *meta* H of C_6_H_5_), 9.65 (d, 1H, *ortho* H of C_5_H_4_N), 7.55 (dd, 1H, *para* H of C_5_H_4_N), 7.15 (dd, 1H, *meta* H of C_5_H_4_N), 7.05 (d, 1H, *meta* H of C_5_H_4_N), 7.15 and 7.35 (s, 4H, *meta* H Mesityl), 4.45 (bs, 4H, CH_2_ of NHC), 2.59, 2.89 and 3.05 (3s, 18H, CH_3_ mesityl), 2.15 (dt, 2H, *iso*-propyl CH of *O*,*N*-ligand), 2.15 (dt, 2H, *iso*-propyl CH of *O*,*N*-ligand), 1.01–1.35 (2d, 6H, *iso*-propyl CH_3_ of *O*,*N*-ligand), 0.10–0.50 (2d, 6H, *iso*-propyl CH_3_ of *O*,*N*-ligand); anal. C_40_H_51_ClN_3_ORu (726.39 g/mol): C 66.14, H 7.08, N 5.78; calcd. for C 65.68, H 6.73, N 5.08.

*Benzylidene-chloro(1,3-bis-(2,4,6-trimethylphenyl)-2-imidazolidinylidene)-[8-quinolinolate]ruthenium* (**12**). A solution of the lithium salt of quinoline (0.99 mmol, 0.150 g) in THF (5 mL) was added dropwise to a solution of **2** (0.24 mmol, 0.200 g) in THF (5–10 mL). Three carbene complexes formed during the synthesis of **12** of which two were completely soluble in pentane, with the third only partially soluble. As a result of the solubility differences of the three carbenes in pentane, two product layers were formed upon slow vacuum condensation. The bottom analytically pure orange-brown layer contained the partially soluble **12** with a H_α_ resonance signal at δ 18.25 ppm (42 mg, 26% yield). ^1^H-NMR (300 MHz, CDCl_3_): δ = 18.25 (s, 1H, Ru=CHPh), 6.85 (d, 2H, *ortho* H of C_6_H_5_), 7.35 (t, 1H, *para* H of C_6_H_5_), 6.72–6.65 (m, 2H, *meta* H of C_6_H_5_), 7.75 (d, 1H, H-1 of naphthyl), 6.40 (d, 1H, H-3 of naphthyl), 6.72–6.65 (m, H-4 of naphthyl), 7.05–6.89 (m, H-5 of naphthyl), 8.85 (d, H-6 of naphthyl), 6.52 and 6.33 (2 bs, 4H, *meta* H of mesityl), 3.95 (bs, 4H, CH_2_ of NHC), 1.95, 2.05 and 2.25 (3s, 18H, CH_3_ of mesityl)*;* anal. C_46_H_47_ClN_3_ORu (678.26 g/mol): C 65.52, H 5.80, N 6.20; calcd. for C 65.04, H 5.40, N 6.13.

### 3.5. General Procedure for the 1-Octene Metathesis Reactions

The metathesis reactions of 1-octene were carried out in a 250 mL three-necked round-bottomed flask fitted with a condenser, thermometer and septum as reported previously [24]. The 1-octene (20 mL) was transferred to the reaction flask and heated to the desired reaction temperature using an oil bath on a controlled hotplate magnetic stirrer. Thereafter the precatalyst amount was added to the flask and the reaction mixture was continuously stirred with a magnetic stirrer bar until the formation of the PMPs was completed. Samples (0.3 mL) were withdrawn with a gastight syringe during the reaction at different time intervals and added to a solution of nonane (0.1 mL), toluene (0.3 mL) and 2 drops of tert-butyl hydrogen peroxide for analysis by GC/FID. Nonane was used as an external standard, toluene to increase sample volume and *tert*-butyl hydroperoxide as a quencher. The experiments were repeated at least 3 times to ensure good reproducibility. Typically the average deviation of the % observed products were 5%. An Agilent 6890 GC equipped with an Agilent 7683 auto-injector, HP-5 capillary column (30 m × 320 µm × 0.25 μm) and a FID was used for analysis. The GC analysis conditions were: inlet temperature 200 °C; N_2_ carrier gas flow rate 94 mL/min; injection volume 0.2 mL (auto injection); split ratio 50:1; oven programming 60 °C for 5min, 60–110 °C at 25 °C/min, 110 °C for 5 min, 110–290 °C at 25 °C/min, 290 °C for 5 min; FID detector temperature 300 °C; H_2_ flow rate 40 mL/min and air flow rate 450 mL/min.

## 4. Conclusions

A number of chelating pyridinyl-alcoholato ligands have been successfully incorporated into **2**, to give a selection of second generation hemilabile precatalysts. Apart from complex **12**, all the complexes showed high activity, temperature stability and a long lifetime towards the self-metathesis of 1-octene, relative to **2**, since after 20 h PMP formation was still visible. Furthermore, **5**, **8**, **10** and **11** showed a 10%–20% higher SMP formation relative to **2**. However, relative to **2**, **9** showed only a 3% increase, **7** showed similar % SMP formation, while a decrease was visible for **6**, demonstrating the improved activity and stability of these complexes. On the other hand, at temperatures ≥ 80 °C, an increase in SMP formation was visible for **6**, **7**, **8** and **9** due to possible precatalyst decomposition or reversible allyl-hydride formation, which is being investigated further.
